# Diabetes-related excess mortality in Mexico: a comparative analysis of national death registries between 2017-2019 and 2020

**DOI:** 10.2337/dc22-0616

**Published:** 2022-10-26

**Authors:** Omar Yaxmehen Bello-Chavolla, Neftali Eduardo Antonio-Villa, Carlos A. Fermín-Martínez, Luisa Fernández-Chirino, Arsenio Vargas-Vázquez, Daniel Ramírez-García, Martín Roberto Basile-Alvarez, Ana Elena Hoyos-Lázaro, Rodrigo M. Carrillo-Larco, Deborah J. Wexler, Jennifer Manne-Goehler, Jacqueline A. Seiglie

**Affiliations:** 1 Research Division, Instituto Nacional de Geriatría; 2 MD/PhD (PECEM) Program, Facultad de Medicina, Universidad Nacional Autónoma de México; 3 Facultad de Química, Universidad Nacional Autónoma de México; 4 Facultad de Medicina, Universidad Nacional Autónoma de México; 5 Department of Epidemiology and Biostatistics, School of Public Health, Imperial College London, London, UK; 6 CRONICAS Centre of Excellence in Chronic Diseases, Universidad Peruana Cayetano Heredia; 7 Harvard Center for Population and Development Studies, Harvard T.H. Chan School of Public Health; 8 Diabetes Unit, Massachusetts General Hospital, Harvard Medical School; 9 Department of Medicine, Harvard Medical School

**Keywords:** Excess mortality, diabetes, COVID-19, health inequalities, glycemic control

## Abstract

**Objective:**

To characterize diabetes-related excess mortality in Mexico in 2020 compared to 2017-2019, during the COVID-19 pandemic.

**Research Design and Methods:**

Retrospective, state-level study using national death registries from Mexican adults ≥20 years for the 2017-2020 period. Diabetes-related death was defined using ICD-10 codes which listed diabetes as the primary cause of death, excluding certificates with COVID-19 as the primary cause of death. Spatial and negative binomial regression models were used to characterize the geographic distribution, socio-demographic and epidemiologic correlates of diabetes-related excess mortality, estimated as increases in diabetes-related mortality in 2020 compared to average 2017-2019 rates.

**Results:**

We identified 148,437 diabetes-related deaths in 2020 (177/100,000 inhabitants), compared with an average of 101,496 deaths in 2017-2019 (125/100,000 inhabitants). In-hospital diabetes-related deaths decreased by 17.8% in 2020 compared to 2017-2019, whereas out-of-hospital deaths increased by 89.4%. Most deaths were attributable to type 2 diabetes (130/100,000 inhabitants). Compared with 2018-2019, hyperglycemic hyperosmolar state and diabetic ketoacidosis were the two contributing causes with the highest increase in mortality (128% and 116% increase, respectively). Diabetes-related excess mortality clustered in southern Mexico and was highest in states with higher social lag, higher rates of COVID-19 hospitalization, and higher prevalence of HbA1c ≥7.5%.

**Conclusions:**

Diabetes-related deaths increased among Mexican adults by 41.6% in 2020 after the onset of the COVID-19 pandemic, occurred disproportionately out-of-hospital, and were largely attributable to type 2 diabetes and to hyperglycemic emergencies. Disruptions in diabetes care and strained hospital capacity may have contributed to diabetes-related excess mortality in Mexico during 2020.

## Introduction

Diabetes mellitus is a leading cause of disability, morbidity, and mortality world-wide. In Mexico, diabetes is the second leading cause of death and has an estimated prevalence of 15.2% (12.8 million adults)^1,2^; furthermore, over the past three decades, mortality attributable to diabetes increased by an alarming 77%^3^. In 2020, during the COVID-19 pandemic, Mexico experienced one of the highest rates of all-cause excess mortality globally (46.5% increase from prior years). Even though the majority of these excess deaths were directly attributable to COVID-19 case mortality, reports by the National Institute for Statistics and Geography (INEGI) suggest that excess deaths in Mexico during 2020 were also attributable to an increase in mortality from non-COVID-19 causes, including cardiovascular disease and diabetes^4^. However, the extent to which diabetes contributed as a cause of this excess mortality during 2020 compared to recent years has not yet been characterized^5^.

Diabetes prevalence, as well as diabetes-related complications and mortality, are tightly associated with socio-demographic inequalities in Mexico^2,6^. These inequalities were unmasked by the COVID-19 pandemic, in part due to a fragmented care infrastructure that preceded COVID-19 and interruptions in in-person care, which disproportionately impacted populations with socioeconomic disadvantage^7,8^. As such, while the intersection of diabetes and COVID-19 and their compounded severity was an important contributor to all-cause excess mortality in Mexico in 2020^9–11^, the pandemic had ripple effects that also impacted care continuity for people with diabetes independent of COVID-19 itself^8^. Furthermore, because hospital saturation was highest in marginalized communities^9,12,13^, we hypothesized that diabetes-related deaths were higher in 2020 compared to prior years and that these excess deaths were associated with higher levels of marginalization. Given that the COVID-19 pandemic is ongoing and continues to pose a significant burden on health systems globally, characterizing the extent to which diabetes-related mortality rates may have increased in 2020 could help guide policies to mitigate interruptions in diabetes care and strengthen existing systems for diabetes care delivery across the healthcare system.

In this study, we sought to characterize: 1) the age-adjusted rates of diabetes-related excess mortality among Mexican adults ≥20 years during 2020, overall and stratified by diabetes type, diabetes-related emergencies and complications as contributing causes of death, and in-hospital vs. out-of-hospital death; 2) the geographic distribution of diabetes-related excess mortality in Mexico; and 3) socio-demographic and epidemiologic correlates of diabetes-related excess mortality in Mexico in 2020.

## Research Design and Methods

### Study design and data source

We conducted a retrospective, state-level study using national death registries obtained from the dynamic information cubes registered by INEGI for the 2017-2020 period, last updated on October 21^st^, 2021. Briefly, INEGI generates annual mortality statistics from death certificates issued by the Ministry of Health. Death registries are comprised of systematic daily mortality records, which are coded using the tenth revision of the International Classification of Diseases (ICD-10)^14^.

### Variables and definitions

#### Outcome variables

Our analysis was centered on two primary outcomes: Diabetes-related mortality and diabetes-related excess mortality.

##### 1) Diabetes-related mortality

Defined according to death certificates generated by INEGI, which listed one of the following ICD-10 codes as the primary cause of death: E10- Type 1 diabetes mellitus, E11-Type 2 diabetes mellitus, and E12-14-Other diabetes mellitus (including malnutrition-related diabetes, other specified diabetes mellitus, and unspecified diabetes mellitus). Diabetes-related mortality was additionally stratified by diabetes-related emergencies and complications listed as contributing causes in each death certificate and defined based on the following ICD-10 codes: hyperglycemic hyperosmolar state (HHS) (E10-14.0), diabetic ketoacidosis (E10-14.1), kidney complications (E10-14.2), ophthalmic complications (E10-14.3), neurological complications (E10-14.4), circulatory complications (E10-14.5), other specified and unspecified complications (E10-14.6 and E10-14.8), multiple complications (E10-14.7), and without complications (E10-14.9). To focus our analysis on diabetes-related mortality only and to exclude the confounding effect of COVID-19 on excess mortality in Mexico^15^, we excluded all death certificates in which COVID-19 was listed as the primary cause of death (ICD-10 code U07.1 or U07.2) during 2020. We also excluded deaths that did not have geographic or socio-demographic information. A flow diagram with study inclusion criteria is provided in Supplementary Figure 1.

##### 2) Diabetes-related excess mortality

**Estimated as the increase in diabetes-related mortality in 2020 compared to the 2017-2019 period average**. This approach is consistent with the definition of excess mortality proposed by Karlinsky and Kobak, which allows for the comparison of all-cause excess mortality across countries and minimizes year to year variations in mortality^16^. Excess deaths were standardized to age-adjusted rates per 100,000 using population age structures by region and state per 5-year increments using population projections provided by the National Population Council^17^. Diabetes-related excess mortality was estimated at the regional level for descriptive purposes and at the state level for modeling (Supplementary Material). **Diabetes-related excess mortality is also presented as percent increase in 2020 compared to the 2017-2019 period**.

#### In-hospital vs. out-of-hospital death

We stratified diabetes-related mortality according to where the death occurred (in-hospital vs. out-of-hospital), based on death certificate information. Out-of-hospital deaths were defined accordingly if the death occurred outside the hospital setting or if the death was coded as occurring at the home of the deceased person or elsewhere (i.e., in the streets in some instances). Deaths without specified place of death were classified as unspecified and were excluded from these analyses (2274 deaths in 2020, 1433 in 2019, 1187 in 2018, and 1206 in 2017). Next, we calculated the ratio of the number of deaths that occurred out-of-hospital divided by those that occurred in-hospital per state and year. We considered the differences in this ratio for 2020 compared to the average ratio in 2017-2019 as the main measure for this outcome.

#### Epidemiologic indicators of diabetes care and diabetes prevalence, as well as COVID-19 seroprevalence

To assess correlates of excess mortality related to the epidemiology of diabetes in Mexico, we analyzed data from the Mexican National Health and Nutrition Survey COVID, which was carried out in 2020 (ENSANUT COVID 2020)^18^. ENSANUT is a population-based survey that aims to evaluate the health and nutritional status of Mexican adults, representative at the national, regional, and rural/urban level. ENSANUT COVID 2020 was conducted from August to November 2020, recruited 24,726 adults ≥20 years, and was used to estimate diabetes-related prevalence and COVID-19 seroprevalence. Diabetes was defined by self-report among individuals who answered “yes” to the question “Has a doctor ever told you that you have diabetes or high blood sugar?” or by either a fasting blood glucose level of ≥126 mg/dL or a hemoglobin A1c (HbA1c) ≥6.5%. Individuals who met the biochemical definition of diabetes but who responded “no” to a prior diagnosis of diabetes were categorized as having undiagnosed diabetes. Suboptimal glycemic management was defined based on an HbA1c level of ≥7.5%^18,19^.

#### Density-independent social lag index

To quantify the state-level impact of sociodemographic inequalities on diabetes-related mortality during 2020, we used the 2020 social lag index (SLI), a composite assessment of the degree of healthcare access, economic well-being, and access to basic services in Mexico^13,20^. Population density was calculated as proposed by INEGI. Because we sought to evaluate inequalities independent of population density, we used residuals of linearly regressed population density onto SLI values to approximate a Density-independent SLI (DISLI), previously validated for Mexico City^12^.

#### State-level epidemiologic indicators of the COVID-19 pandemic

To evaluate the association between COVID-19 and diabetes-related excess mortality, we also included variables of relevance to COVID-19 epidemiology in Mexico. State-wide incidence, hospitalization, and deaths attributable to COVID-19 in Mexico were evaluated using data from the General Directorate of Epidemiology of the Mexican Ministry of Health, which is an open-source dataset that provides daily updated information of suspected COVID-19 cases^9^. COVID-19 cases are confirmed with a positive RT-PCR or rapid antigen test for SARS-CoV-2^9,13,21^. Estimates were obtained by COVID-19 cases with comorbid diabetes. All metrics were weighted to their respective population by state to reflect rates per 100,000 inhabitants using data from National Population Council of Mexico (CONAPO).

### Statistical Analysis

First, to visualize differences in diabetes-related mortality in the 2017-2020 period, we plotted diabetes-related deaths per 100,000 inhabitants by month of occurrence, overall and stratified by diabetes type. We also plotted the overall number of diabetes-related deaths, stratified by diabetes type, age group, and out-of-hospital vs. in-hospital setting, and compared the 2020 estimates to the average in 2017-2019. We then disaggregated the rates of diabetes-related mortality per 100,000 inhabitants by diabetes-related complications listed as contributing causes of death in the 2018-2020 period. The year 2017 was excluded due to incomplete ICD-10 codes for emergencies and complications related to diabetes-related deaths. This analysis was also stratified by out-of-hospital vs. in-hospital mortality. **Diabetes complications listed as contributing causes of death were also classified as acute (HHS and diabetic ketoacidosis) or chronic (kidney, ophthalmic, neurological, and circulatory) complications** (Supplementary Figure 4).

Second, to visualize the geographical distribution of diabetes-related excess deaths in Mexico, we used chloropleth maps with the *ggmap* R package with the quantile method. To evaluate the spatial dependence of diabetes-related excess mortality and the out-of-hospital to in-hospital death ratio, we used Moran’s I statistic, which was obtained as an indicator of global spatial autocorrelation, and its significance was assessed through an inference technique based on randomly permuting the observed values over the spatial units. We also evaluated hotspots of excess mortality and the out-of-hospital to in-hospital death ratio to confirm autocorrelation using the Getis-Ordi Gi statistic.

Next, we used spatial autocorrelation to characterize whether key indicators relevant to diabetes care and diabetes prevalence in Mexico were associated with diabetes-related excess mortality. Similarly, we examined whether socio-demographic inequalities as proxied by DISLI, and COVID-19 indicators, were associated with age-adjusted diabetes-related excess mortality. Bivariate correlations between epidemiological indicators and age-adjusted diabetes-related excess mortality were evaluated with Lee’s L test for spatial autocorrelation using spatial weights matrix within the *spdep* R package^22^. We also assessed the correlation of these epidemiological indicators with the out-of-hospital to in-hospital death ratio, as a proxy of their impact on access to medical care.

Finally, the simultaneous impact of all evaluated indicators on age-adjusted diabetes-related excess mortality was analyzed using negative binomial regression, with log-transformed population of each state as the regression offset. We used the Global Moran I test for regression residuals for all models and identified no significant spatial autocorrelation; therefore, we fitted all models without incorporating spatial effects. All statistical analyses were conducted using R software version 4.1.2.

## Results

### Diabetes-related mortality in Mexico in 2020 compared to 2017-2019

**Figure 1:**
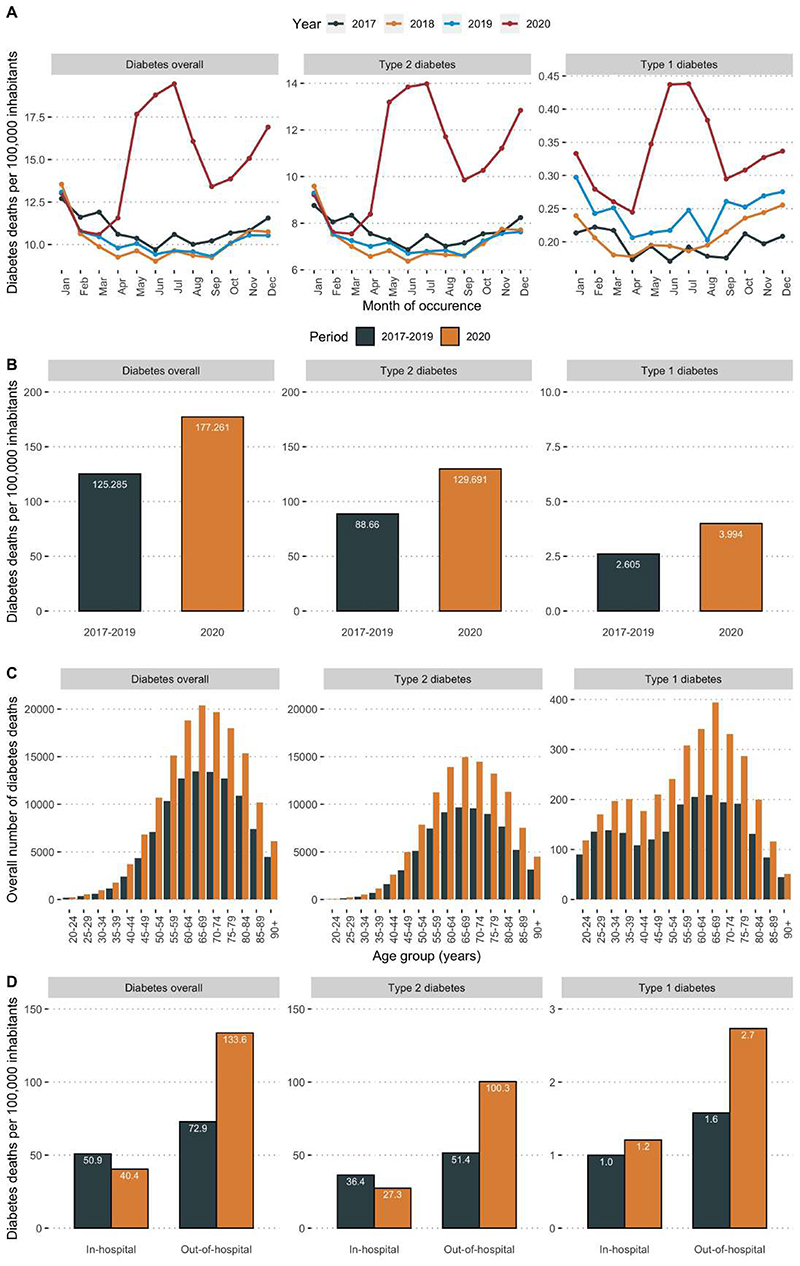
Monthly trends over time of diabetes-related deaths per 100,000 inhabitants during the 2017-2020 period and stratified by overall, type 1 and type 2 diabetes classified using ICD-10 criteria (A). We also show a comparison of diabetes-related deaths between the average of 2017-2019 and 2020 by diabetes type (B), by age group per 5-year increments (C), and stratified according to out-of-hospital vs. in-hospital mortality (C).

We analyzed data from 452,924 diabetes-related deaths in Mexico during the 2017-2020 period. We identified 148,437 diabetes-related deaths (177 per 100,000 inhabitants) in 2020, compared with an average of 101,496 deaths in 2017-2019 (125 per 100,000 inhabitants); a 41.6% increase in diabetes-related deaths in 2020 compared to the average reported in the 2017-2019 period (Figure 1). Overall, the highest rate of diabetes-related mortality occurred after the month of May, with a peak during the June-July period after the onset of the COVID-19 pandemic (Figure 1A); notably these trends closely followed COVID-19 related mortality trends (Supplementary Figure 2). When stratified by diabetes type, the largest share of diabetes-related deaths in 2020 was attributable to type 2 diabetes (130 per 100,000 inhabitants), and type 1 diabetes (3.99 per 100,000 inhabitants). Compared with diabetes-related deaths in 2017-2019, these rates correspond to an excess mortality of 46.7% for type 2 diabetes and 53.5% for type 1 diabetes (Figure 1B). Diabetes-related mortality was higher in 2020 than during the 2017-2019 period across all ages for all diabetes types (Figure 1C). Regarding place of death, out-of-hospital diabetes-related deaths increased by 89.4%, from an average of 59,061 diabetes-related deaths (50.9 per 100,000 inhabitants) during 2017-2019 to 111,870 deaths (133.6 per 100,000 inhabitants) in 2020. Conversely, in-hospital deaths decreased by 17.8%, from 41,176 diabetes-related deaths (50.9 per 100,000 inhabitants) in 2017-2019 to 33,825 deaths (40.4 per 100,000 inhabitants) in 2020. This trend was particularly pronounced for type 2 diabetes-related deaths, with a decrease in hospital deaths of 10.5% and an increase in out-of-of hospital deaths of 195%. Compared to 2017-2019, the out-of-hospital to in-hospital death ratio increased from 1.43 to 3.31 for all diabetes related-deaths (Figure 1D), from 1.41 to 3.67 for type 2 diabetes-related death (Figure 1B), from 1.60 to 2.25 for type 1 diabetes-related deaths (Figure 1C), and from 1.47 to 2.58 for other diabetes-related deaths (Supplementary Figure 3).

### Diabetes-related mortality in 2020 compared with 2017-2019, according to diabetes-related emergencies and complications as contributing causes of death

**Figure 2:**
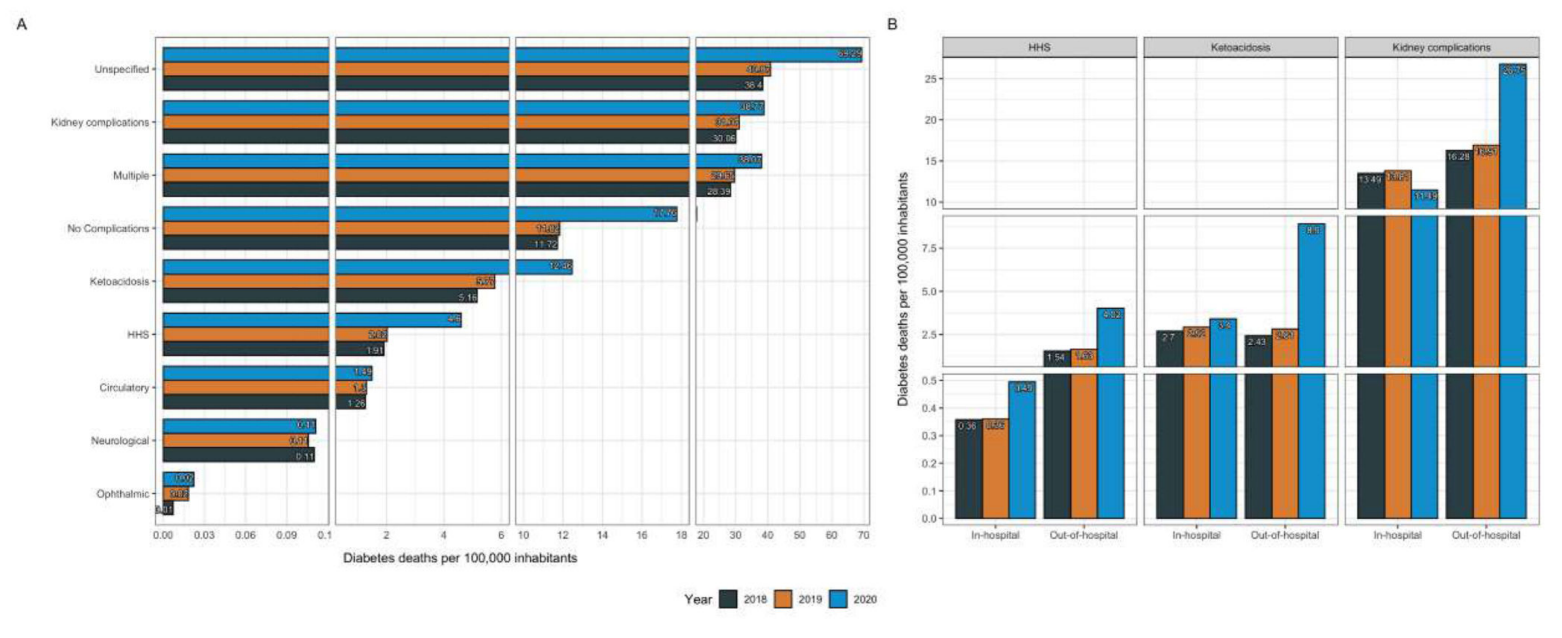
Diabetes-related mortality rates standardized per 100,000 population and disaggregated by diabetes-related emergencies and complications as contributing causes of death in 2018-2019 compared to 2020 (A). Contributing causes of death were classified using ICD-10 codes for each specific emergency and complication. The figure also shows the three leading diabetes-related emergencies and complications as contributing causes of death according to out-of-hospital vs. in-hospital mortality in 2020 compared to 2018-2019 (B). **Abbreviations:** HHS, Hyperglycemic Hyperosmolar State

The stratification of diabetes-related mortality according to diabetes-related emergencies and complications as a contributing cause of death is presented in Figure 2. **Overall, the highest relative increase in mortality for diabetes-related complications was observed for acute complications (131.8% increase), followed by chronic complications (36.4% increase) as seen in** Supplementary Figure 4. Compared with 2018-2019, HHS (128% absolute increase) and diabetic ketoacidosis (116% absolute increase) were the two contributing causes with the highest associated increase in diabetes-related deaths (Figure 2A). Other diabetes-related complications that increased compared to prior years were unspecified complications (69.5%), followed by kidney complications (24.9%), ophthalmic complications (22.2%), and lower-limb circulatory complications (15.1%). Notably, diabetes-related deaths with multiple complications and without complications also increased by 28.4% and 50.2%, respectively (Figure 2A). When stratified by place of death, we observed an increase in both in-hospital and out-of-hospital deaths for HHS (37.9% and 154% increase, respectively) and diabetic ketoacidosis (20.9% and 240% increase, respectively), while for kidney-related complications, we observed a 13.5% decrease in in-hospital and a 61.2% increase in out-of-hospital deaths (Figure 2B). **Stratified analyzes that include all diabetes-related complications as contributing causes of death according to diabetes type are provided in** Supplementary Figure 5. **Overall, we observed that mortality attributable to diabetes-related complications increased consistently for all diabetes types and was primarily attributable to HHS, diabetic ketoacidosis, and kidney complications**.

### Geographic distribution and correlates of diabetes-related excess mortality in Mexico

**Figure 3:**
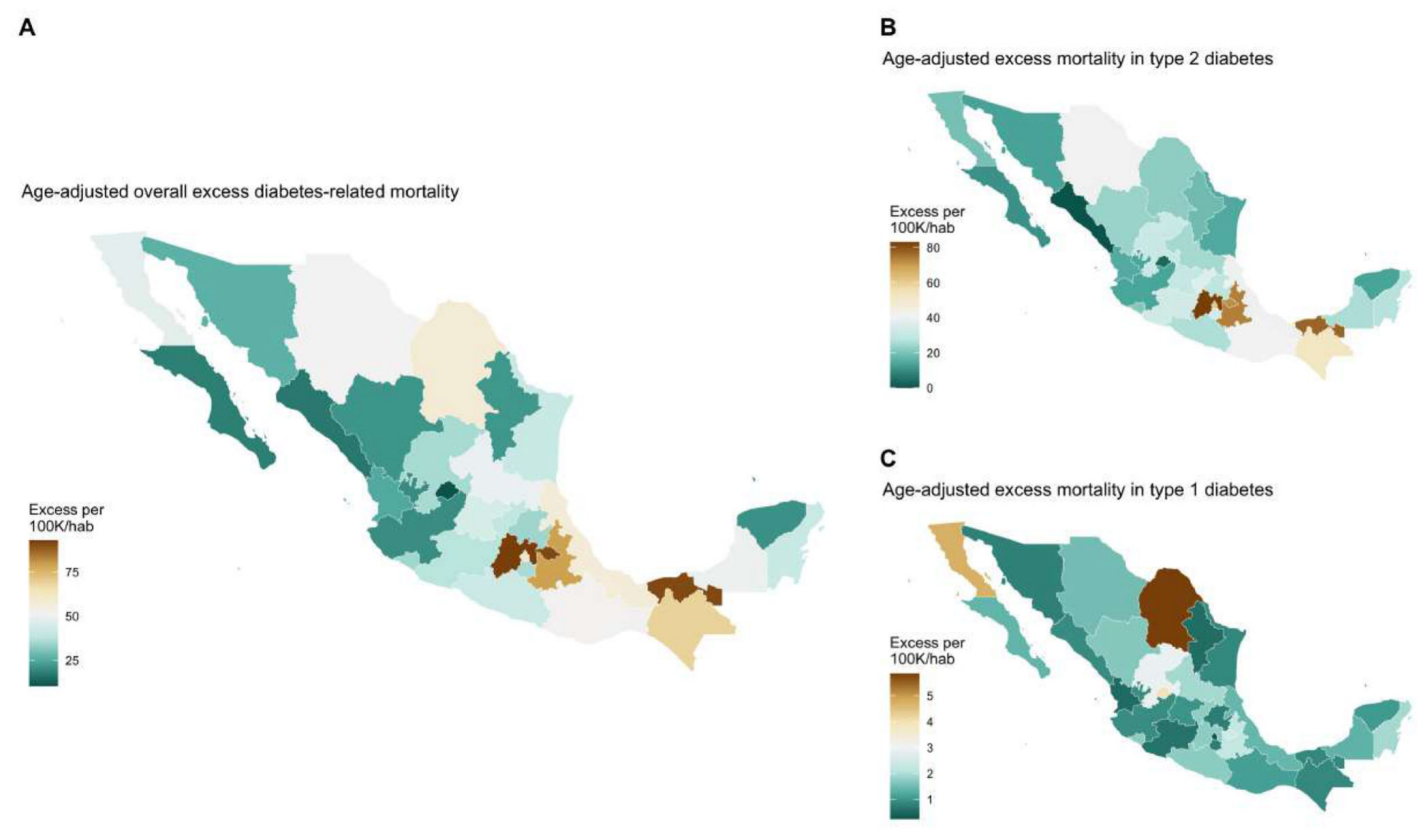
Choropleth maps showing the distribution of estimated age-adjusted diabetes-related excess mortality in Mexico during 2020 compared to 2017-2019, overall (A) and stratified by type 2 (B), and type 1 diabetes (C), according to ICD-10 criteria. Distribution of all evaluated measures was estimated using the quantile method with the *biscale* R package.

We observed spatial autocorrelation in the geographic distribution of diabetes-related excess deaths in Mexico in 2020 compared to 2017-2019 (Moran’s I=0.335, p=0.002, Figure 3). Diabetes-related excess mortality clustered near the Southeast and Gulf of Mexico regions, as identified using local indicators of spatial autocorrelation with Moran’s local statistic and the Getis-Ordi Gi statistic (Supplementary Figure 6). Similarly, the out-of-hospital to in-hospital mortality ratio increased markedly in 2020 compared to 2017-2019 and displayed a strong spatial autocorrelation that clustered within the same region (Moran’s I=0.481, p<0.001). This increase was primarily attributed to higher out-of-hospital diabetes-related mortality for type 2, but not for type 1 diabetes (Supplementary Figure 7).

**Figure 4:**
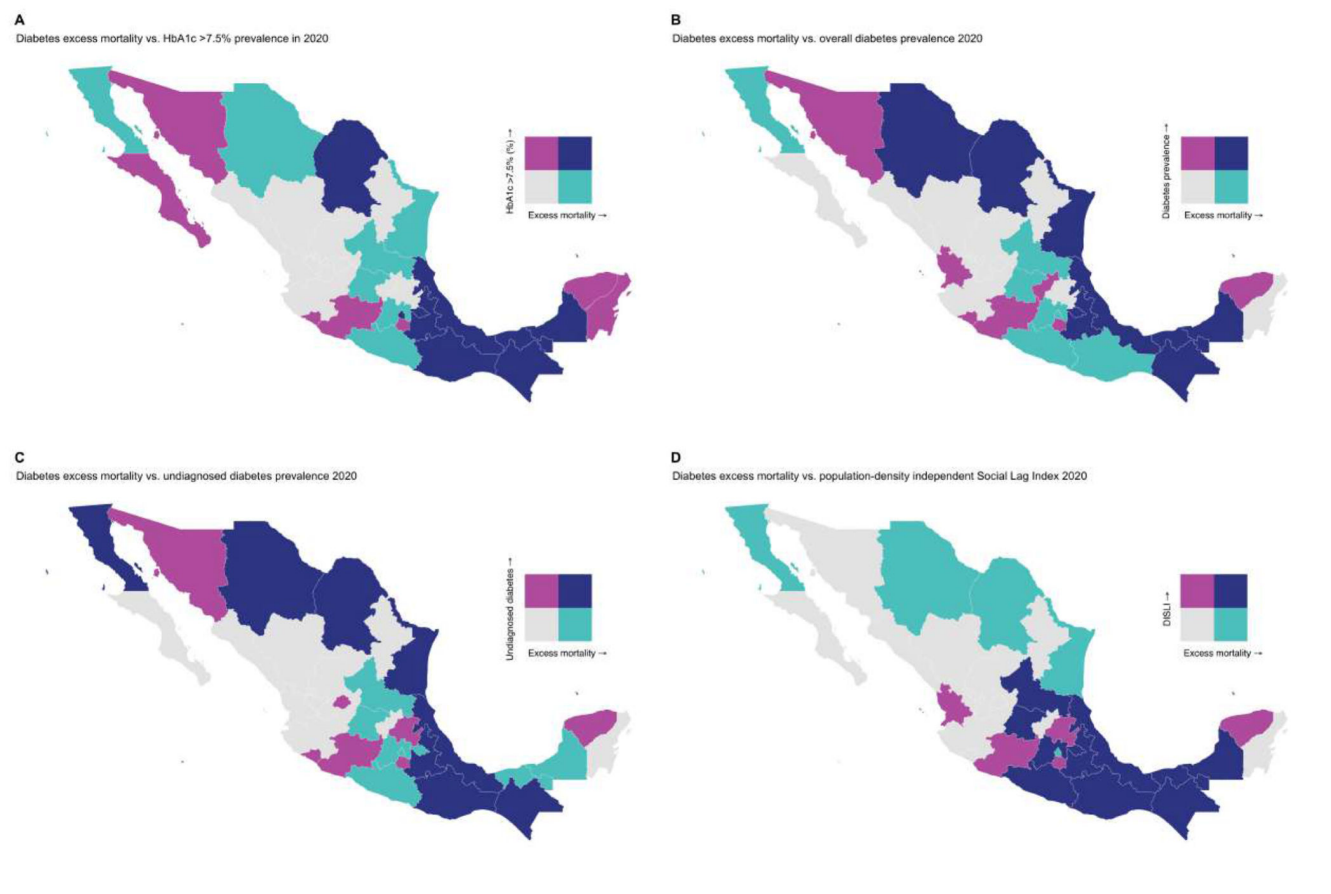
Bivariate choropleth maps showing the geographical distribution of high age-adjusted diabetes-related excess mortality in Mexico with epidemiological indicators related to diabetes care in 2020, including prevalence of HbA1c ≥7.5% (A), diabetes prevalence (B), undiagnosed diabetes prevalence (C), and the 2020 population density-independent social lad index (DISLI, D). Distribution of all evaluated measures was estimated using the quantile method with the *biscale* R package.

We also identified a spatial correlation between diabetes-related excess mortality and the DISLI index of marginalization (Lee’s L statistic= 0.326, p<0.001, Figure 4). Higher DISLI was also correlated with high prevalence of HbA1c levels ≥7.5% (Lee’s L statistic= 0.326, p<0.001). Furthermore, we identified a strong spatial correlation for the out-of-hospital to in-hospital mortality ratio with DISLI (Lee’s L statistic= 0.380, p<0.001) and HbA1c >7.5% prevalence (Lee’s L statistic= 0.247, p=0.047, Supplementary Figure 8). There was no spatial correlation between diabetes-related excess mortality and COVID-19 indicators (Supplementary Figure 9).

### Association between epidemiologic indicators and diabetes-related excess mortality

Age-adjusted diabetes-related excess mortality was highest in states with higher DISLI (IRR 1.18, 95%CI 1.01-1.37), higher log-transformed rates of COVID-19 hospitalization (IRR 1.28, 95%CI 1.06-1.55), and higher prevalence of HbA1c ≥7.5% (IRR 1.04, 95%CI 1.01-1.07), as shown in Supplementary Table 2. The association between higher DISLI and higher diabetes-related excess mortality was particularly pronounced for type 2 diabetes (IRR 1.32, 95%CI 1.08-1.63), which was also associated with higher rates of COVID-19 hospitalization (IRR 1.32, 95%CI 1.01-1.71). For other diabetes types, age-adjusted diabetes-related excess mortality was associated with higher regional COVID-19 seroprevalence (IRR 1.02, 95%CI 1.01-1.04), and higher overall prevalence of diabetes (IRR 1.04, 95%CI 1.00-1.07).

## Conclusions

**In this study of diabetes-related mortality in Mexico, we identified 452,924 diabetes-related deaths among Mexican adults in 2020, which corresponds to a 41.6% increase in diabetes-related mortality, after excluding COVID-related death, in 2020 compared to the 2017-2019 period. Excess deaths were largely attributable to type 2 diabetes and to hyperglycemic emergencies and occurred disproportionately out-of-hospital, with an 89.4% increase in out-of-hospital diabetes-related deaths in 2020 compared to the 2017-2019 period. Diabetes-related excess mortality clustered in southern Mexico, was associated with higher state-level marginalization, higher rates of COVID-19 hospitalizations, and higher prevalence of suboptimal glycemic management. These findings highlight the dramatic increase in diabetes-related mortality that occurred in excess in Mexico in 2020 compared to prior years. Disruptions in diabetes care and strained hospital capacity due to the COVID-19 pandemic may have contributed to diabetes-related excess mortality in Mexico in 2020**.

We also report a substantial increase in diabetes-related emergencies and complications as contributing causes of death in 2020 compared to 2017-2019. Alarmingly, deaths attributable to HHS and diabetic ketoacidosis increased by more than 2-fold in 2020 compared to 2018-2019, with this increase observed predominantly in the out-of-hospital setting (154% increase for HHS and 240% increase for diabetic ketoacidosis). These findings are particularly concerning given that HHS and diabetic ketoacidosis are generally readily treatable conditions, with mortality rates having dropped substantially with access to inpatient care^23,24^. Other diabetes-related microvascular complications as contributing causes of death also increased in 2020 compared to prior years, particularly renal, ophthalmic, and circulatory complications. Given pandemic-related interruptions in care, delayed access to glucose-lowering medications, including insulin, may have contributed to worsening glycemic management and an overall increase in diabetes-related complications^8^. Overall, these findings suggest that interruptions in diabetes care in the setting of the COVID-19 pandemic may have impacted both the acute care of life-threatening diabetes complications as well as the management of chronic complications, such as the need for renal replacement therapy.

The geographic distribution of diabetes-related excess mortality in Mexico in 2020 was subject to regional differences, with higher rates of diabetes-related excess mortality in the central and southeast regions of Mexico. These excess deaths were associated with higher socio-demographic inequalities (as proxied by DISLI), higher rates of suboptimal glycemic management, and increased rates of COVID-19 hospitalizations in 2020. An increase in out-of-hospital and a decrease in in-hospital diabetes-related mortality was also observed in these regions. Similarly, we identified a cluster of significantly higher diabetes-related excess mortality and large increases in the ratio of out-of-hospital to in-hospital mortality in the southeast region of Mexico; notably, this region displayed a spatial correlation between excess diabetes-related out-of-hospital deaths and both DISLI and increased rates of suboptimal glycemic management, suggesting that this region was particularly vulnerable to socio-demographic inequalities in diabetes care. The regional differences in diabetes-related excess mortality for type 1 diabetes (highest in northern Mexico) compared to those observed for type 2 diabetes (highest in southern Mexico) mirror the regional differences in prevalence for each condition^2,19,25,26^.

Our findings expand the existing literature on excess mortality documented in the context of the COVID-19 pandemic and provide a unique focus on cause-specific excess mortality attributed to high-burden cardio metabolic conditions in Mexico^15^. Only one prior study conducted in Mexico has documented diabetes-related mortality, but those estimates preceded the COVID-19 pandemic^3^. In particular, our findings contribute to existing literature that suggests that the COVID-19 pandemic interfered with routine diabetes care in Mexico, particularly in the southern region, which has a higher prevalence of suboptimal glycemic management^9,11,12,19^. These findings are particularly important when considering that the southern region of Mexico faces increased socio-economic inequalities, including a limited healthcare infrastructure, which may have impacted quality and access to care for chronic diseases, including diabetes^27^. Since increasing rates of infections and hospital saturation were observed in later COVID-19 waves, a sustained increase in diabetes-related excess mortality and diabetes complication is possible and should be explored in future analyses. Special efforts should be sought by Mexican public health authorities to reduce health inequalities within this region, and to strengthen care for diabetes and other cardio-metabolic diseases.

Mechanisms underlying the impact of socio-demographic inequalities on diabetes care and diabetes-related excess mortality in Mexico are not well understood and require further study. The epidemiology of diabetes in Mexico is complex and heterogeneous, with data showing a large proportion of diabetes cases relating to obesity and glucotoxicity-mediated decreased β-cell function^28^. Socio-demographic inequalities also markedly impact diabetes care for both type 1 and type 2 diabetes^28–31^. The combined influence of these inequalities likely worsens glycemic management and increases morbidity and mortality in patients living with diabetes. These factors, combined with the ongoing COVID-19 pandemic, which led to hospital saturation in areas with high population density and high social lag, may have contributed to diabetes-related excess mortality in Mexico^32–34^.

## Limitations

Our study has some strengths and limitations. The strengths of our analysis include the use of several nationally representative datasets, which allowed us to gain insight into potential socio-demographic, epidemiologic, and COVID-19-related correlates of diabetes-related excess mortality in Mexico. Second, we standardized all mortality analyses by age, which allowed for adequate comparisons across Mexican regions with a diverse population structure and more precise estimation of diabetes-related excess mortality. Finally, given that the pandemic had a differential impact across Mexico, we explored spatial effects in the influence of all evaluated epidemiological indicators; this allowed us to understand the regional impact of diabetes-related excess mortality to better inform public policy. We also acknowledge the following limitations, which should prompt caution in the interpretation of our results. First, the case definition for diabetes-related death and acute and chronic diabetes complications as contributing causes of death was derived from ICD-10 codes, as opposed to more granular clinical data, and thus is subject to a degree of diagnostic uncertainty and possible misclassification, particularly for deaths that occurred outside of the hospital. However, the International Classification of Diseases that generates the ICD-10 codes was designed to promote international comparability in the collection, processing, classification, and presentation of mortality statistics^14^. Of note, mortality statistics based on ICD-10 codes are routinely used in the ascertainment of mortality trends in epidemiological research studies^35–37^. Second, by using state-level variables, we were unable to perform inferences for all identified associations at individual or even local levels; this is particularly relevant for socio-demographic inequalities, indicators of glycemic control (HbA1c), and COVID-19 seroprevalence, which may have significant heterogeneity within Mexican states at the municipal, local, and individual levels. Finally, since ascertainment of COVID-19 cases in Mexico has been insufficient^21^, many SARS-CoV-2 infections could have been undetected and some of these may have led to diabetes-related complications and deaths such as diabetic ketoacidosis or HHS^38,10^. Therefore, we cannot rule out that a portion of excess mortality formally ascribed to diabetes could have been in fact attributable to COVID-19, despite the fact that ascertainment by INEGI is generally robust^39^. Moreover, though we excluded cases with COVID-19 as the primary cause of death and centered our analysis on cases with diabetes as the primary cause of death, it is possible that COVID-19 was a contributor to diabetes-related mortality given what has been extensively documented on the compounded severity of these two conditions.

### Future directions

**Future studies that include individual-level data with higher granularity of clinical variables are needed to further understand the excess mortality data presented in this study and to identify specific areas of intervention to prevent increases in diabetes-related mortality**.

In conclusion, we report a 41.6% increase in diabetes-related mortality in Mexico in 2020 among adults 20 years and older, after excluding COVID-related death, compared to the 2017-2019 period. Excess deaths were largely attributable to type 2 diabetes and to hyperglycemic emergencies, occurred disproportionately out-of-hospital, clustered in southern Mexico, and were associated with higher state-level marginalization, higher rates of COVID-19 hospitalizations, and higher prevalence of suboptimal glycemic management. Diabetes-related excess mortality was also observed for type 1 diabetes, with excess mortality regionally concentrated in northern Mexico, mirroring the epidemiological distribution of type 1 diabetes prevalence in Mexico. Our findings suggest that readily treatable, high morbidity diabetes-related conditions were likely untreated due to the constraints of the health care system during the COVID-19 pandemic in Mexico. Policies aimed at strengthening hospital capacity and mitigating disruptions in care for diabetes and diabetes-related emergencies should be prioritized to minimize preventable mortality from diabetes and diabetes-related complications.

## Supplementary Material

Supplementary Material

## Data Availability

All code, datasets and materials are available for reproducibility of results at https://github.com/oyaxbell/diabetes_excess/
