# Supplementary material for: Diabetes-related excess mortality in Mexico: a comparative analysis of national death registries between 2017-2019 and 2020
Source: Diabetes Care. Author manuscript; Available in PMC 2022 Dec 1. (PMC7613876; doi:10.2337/dc22-0616)
Supplement: Supplementary Material [file EMS156161-supplement-Supplementary_Material.pdf]

SUPPLEMENTARY MATERIAL

Diabetes-related excess mortality in Mexico: a comparative analysis of national death registries between 2017-2019 and 2020

Omar Yaxmehen Bello-Chavolla, Neftali Eduardo Antonio-Villa, Carlos A. Fermín-Martínez, Luisa Fernández-Chirino, Arsenio Vargas-Vázquez, Daniel Ramírez-García, Martín Roberto Basile-Alvarez, Ana Elena Hoyos-Lázaro, Rodrigo M. Carrillo-Larco, Deborah J. Wexler, Jennifer Manne-Goehler, Jacqueline A. Seiglie

SUPPLEMENTARY RESULTS

Geographic variability in age-adjusted diabetes-related excess mortality in Mexico

Overall, age-adjusted diabetes-related mortality was highest in Mexico State, Tabasco, and Tlaxcala, with a cluster of high diabetes-related excess mortality located in the Southeast of Mexico. When stratified by diabetes type, type 2 diabetes-related excess mortality mirrored that of overall diabetes-related excess mortality, while type 1 diabetes-related excess mortality was highest in the northern states of Baja California, Chihuahua, and Aguascalientes (**Figures 3A-C**). Mortality for other diabetes types was highest in the northern states of Baja California, Coahuila, and Tamaulipas. In bivariate analyses, we identified that a cluster of states with high age-adjusted diabetes-related excess mortality and high prevalence of HbA1c levels ≥7.5% was observed in the Southeast region of Mexico and in the state of Chihuahua, with a similar trend observed for diabetes prevalence (**Figures 3D-F**).

SUPPLEMENTARY FIGURES

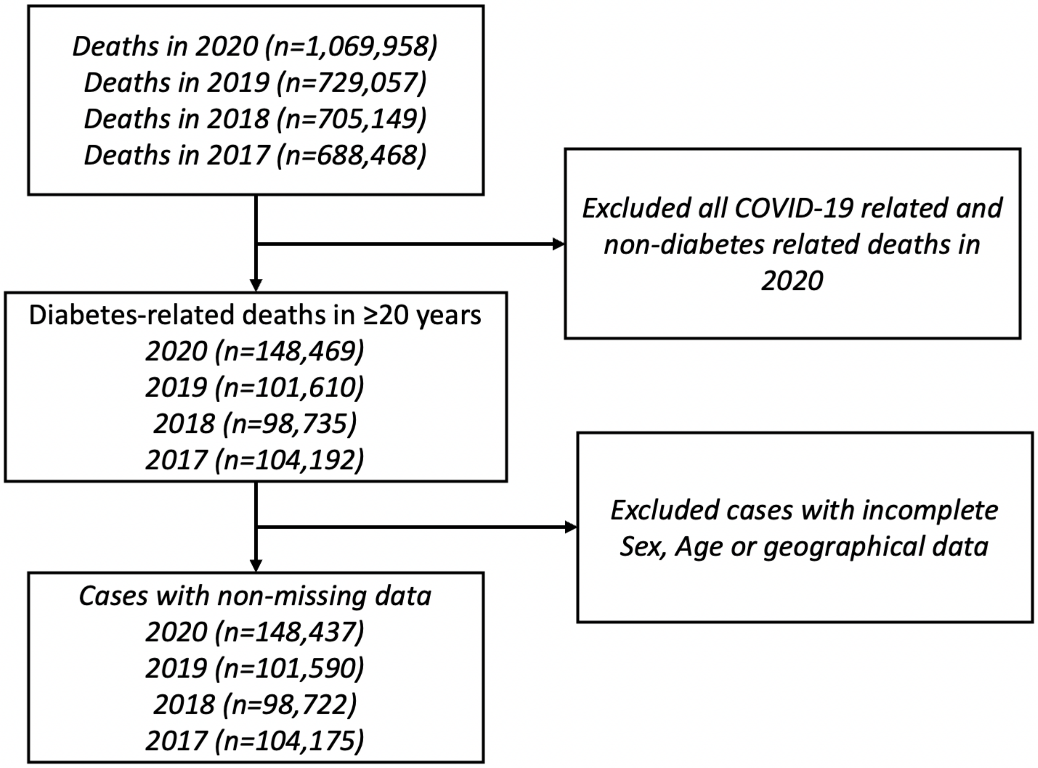

Supplementary Figure 1. Flowchart diagram of data selection for the study in the mortality dataset from 2017-2020.

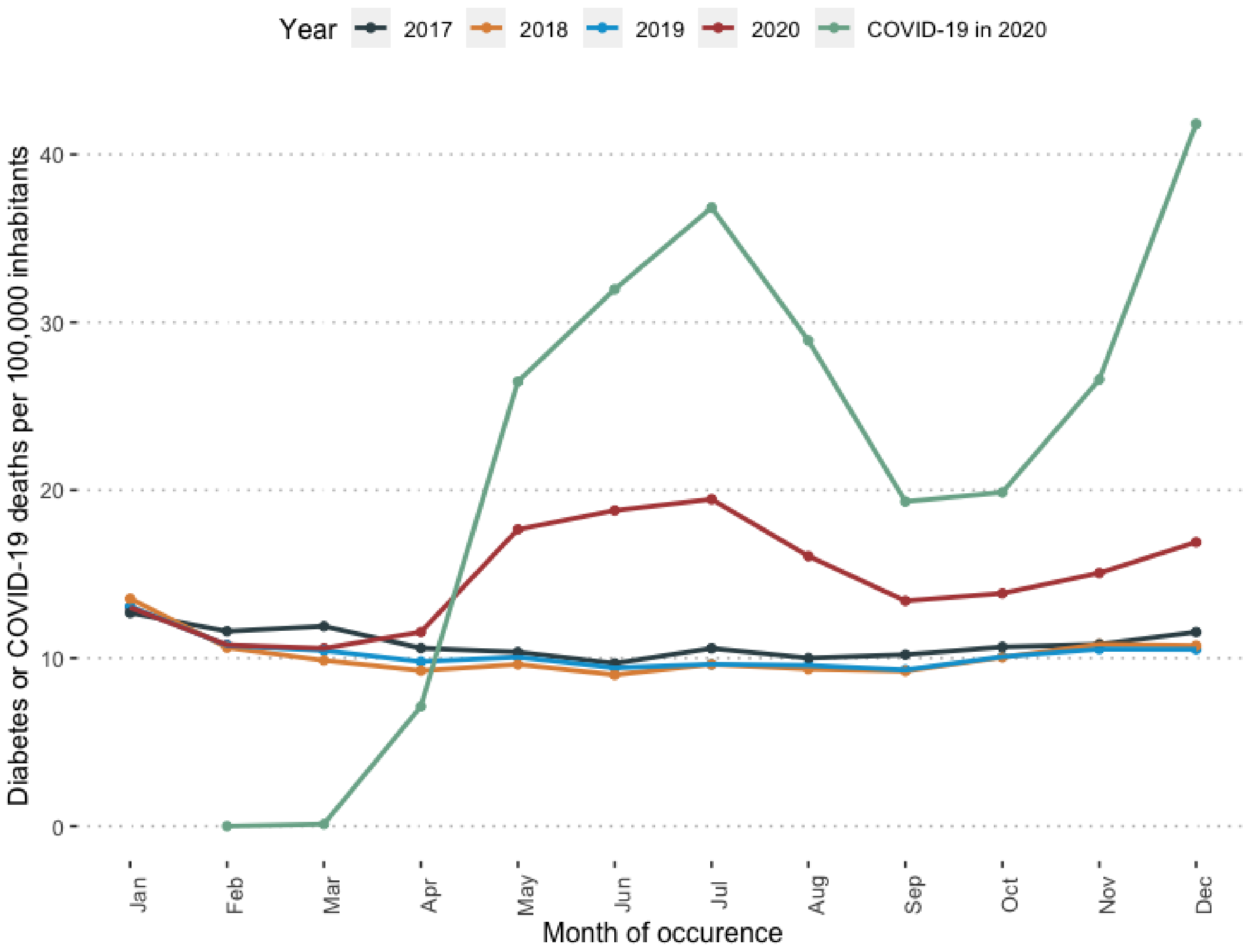

Supplementary Figure 2. Diabetes-related mortality in the 2017-2020 period (ICD-10 codes E10-E14) compared to COVID-19 related deaths in 2020 (ICD-10 codes U07.1 U07.2).

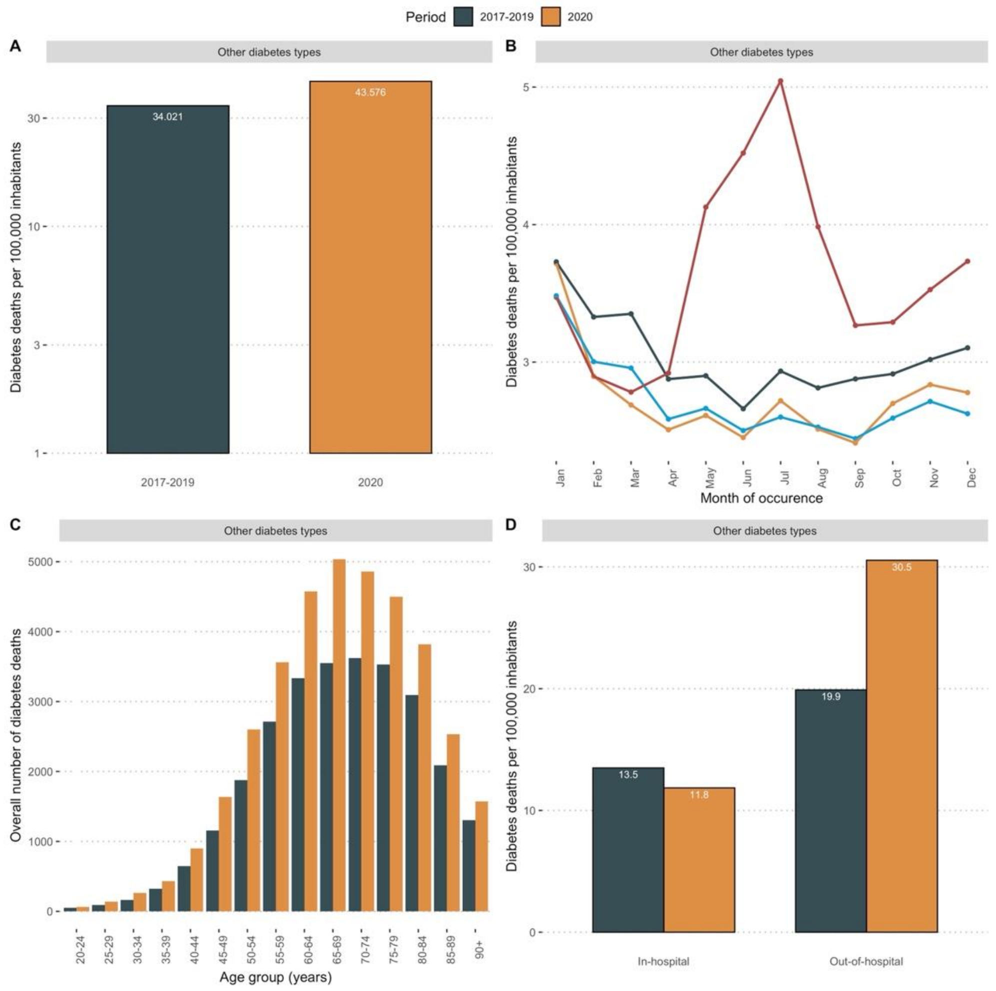

Supplementary Figure 3. Diabetes-related mortality for other diabetes types during the 2017-2019 period compared to 2020 (A) and stratified by month (B), age group by 5-year increments (C) and in-hospital vs. out-of-hospital setting (D).

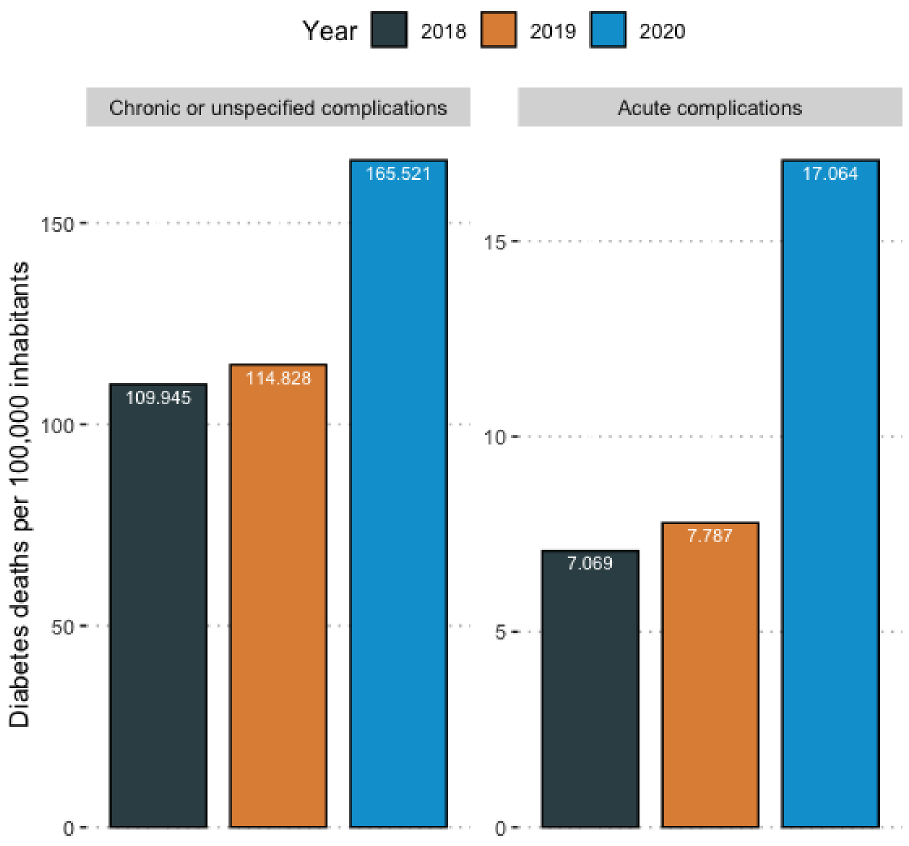

Supplementary Figure 4. Diabetes-related mortality in Mexico during 2018, 2019 and 2020 stratified by diabetes-related complications which contributed as causes of death. Acute diabetes complications include hyperglycemic hyperosmolar state and ketoacidosis, whilst chronic and unspecified complications include all other categories listed in the Methods section of the manuscript.

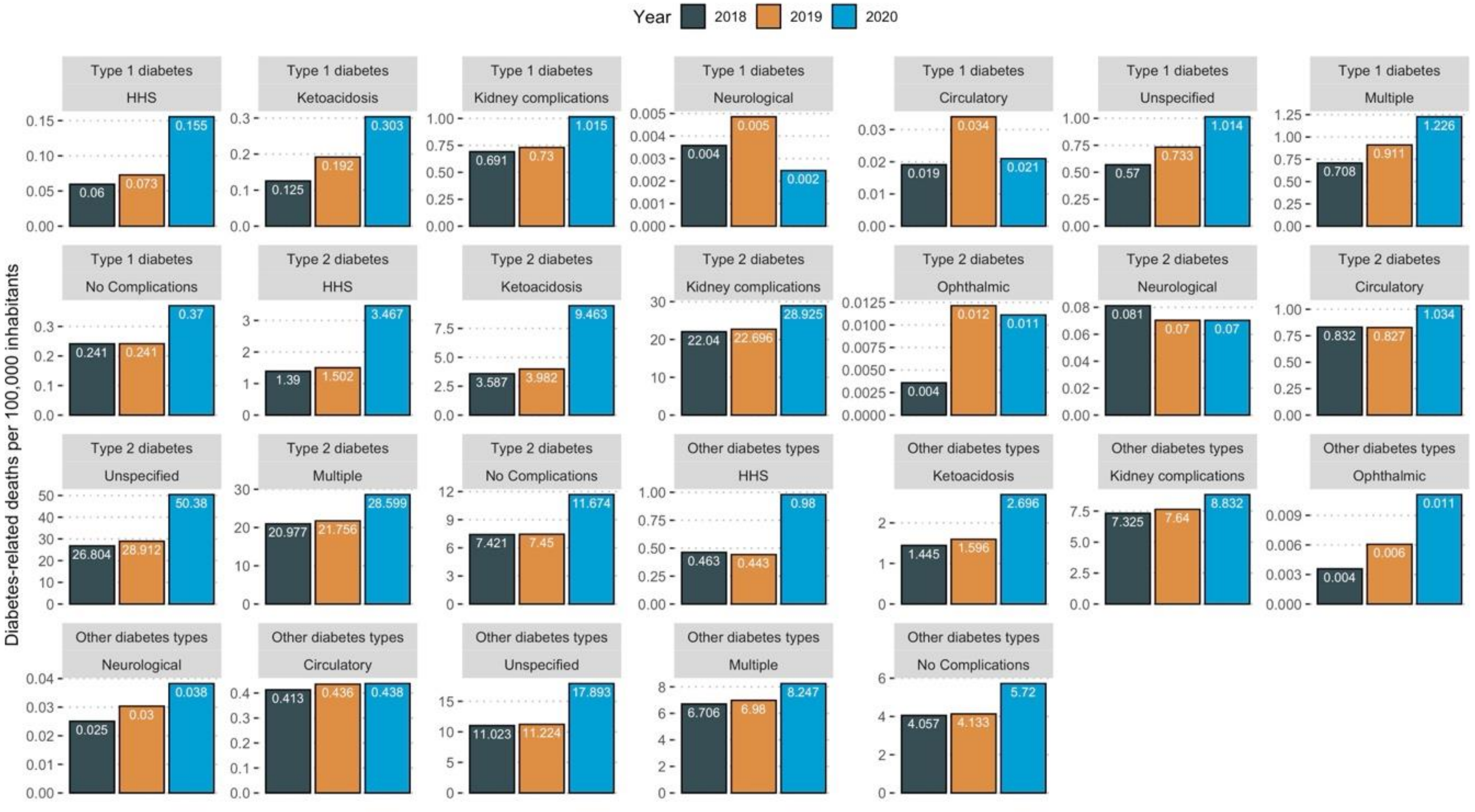

Supplementary Figure 5. Mortality rates for diabetes-related emergencies and complications as contributing causes of death in the 2018-2020 period, stratified by diabetes type, standardized to 100,000 inhabitants. Deaths were categorized using ICD-10 codes.

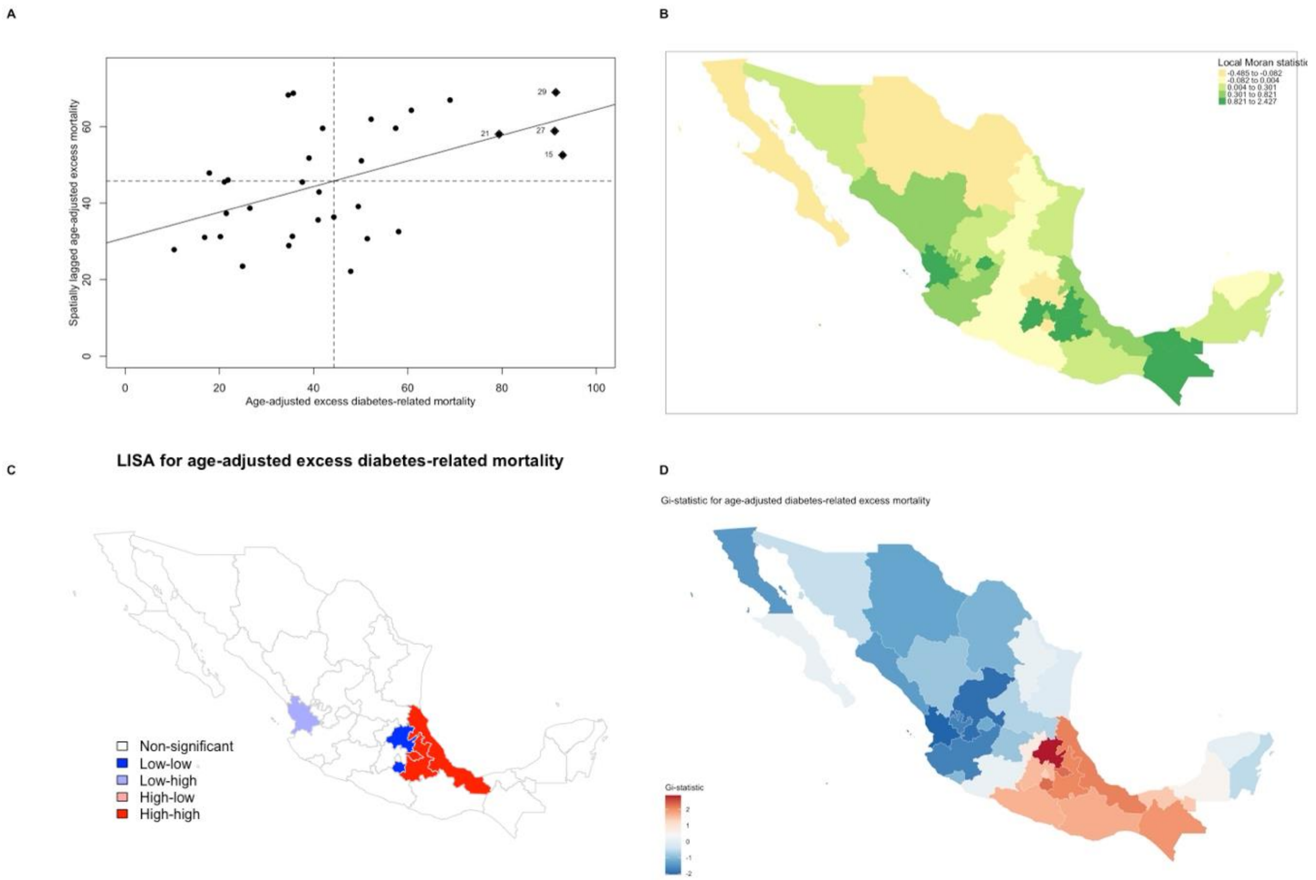

Supplementary Figure 6. Spatial autocorrelation of age-adjusted excess diabetes-related mortality in Mexico using Moran's I statistic (A). Figure also shows the distribution of Local Moran Statistic (B) and the local indicators of spatial autocorrelation (LISA) using Moran's I statistic for age-adjusted diabetes-related excess mortality. Panel C shows a cluster of states high diabetes-related excess mortality surrounded by similar states in the Gulf of Mexico Region (C). Panel D shows the distribution of the Getis-Ordi Gi statistic, representing hot spots of diabetes-related excess mortality primarily located in southern Mexico and the Gulf region (D).

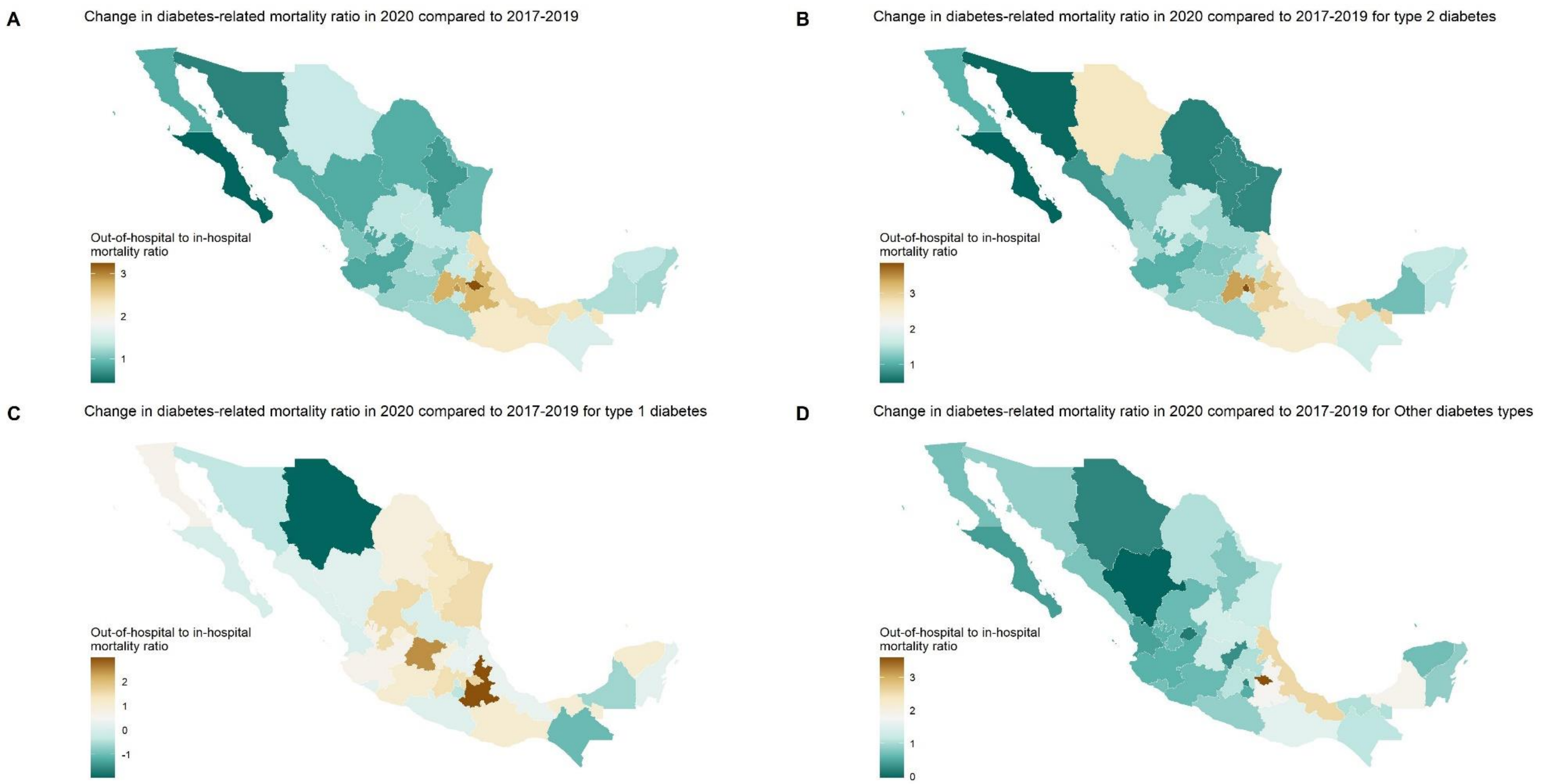

Supplementary Figure 7. Chloropleth maps showing the geographical distribution of the difference in out-of-hospital to in-hospital death ratio in 2020 compared to the average of 2017-2019 for overall diabetes-related mortality (A), and stratified for type 2 (B), type 1(C) and other types of diabetes (D), codified using ICD-10 causes of death registered by INEGI and the Mexican Ministry of Health.

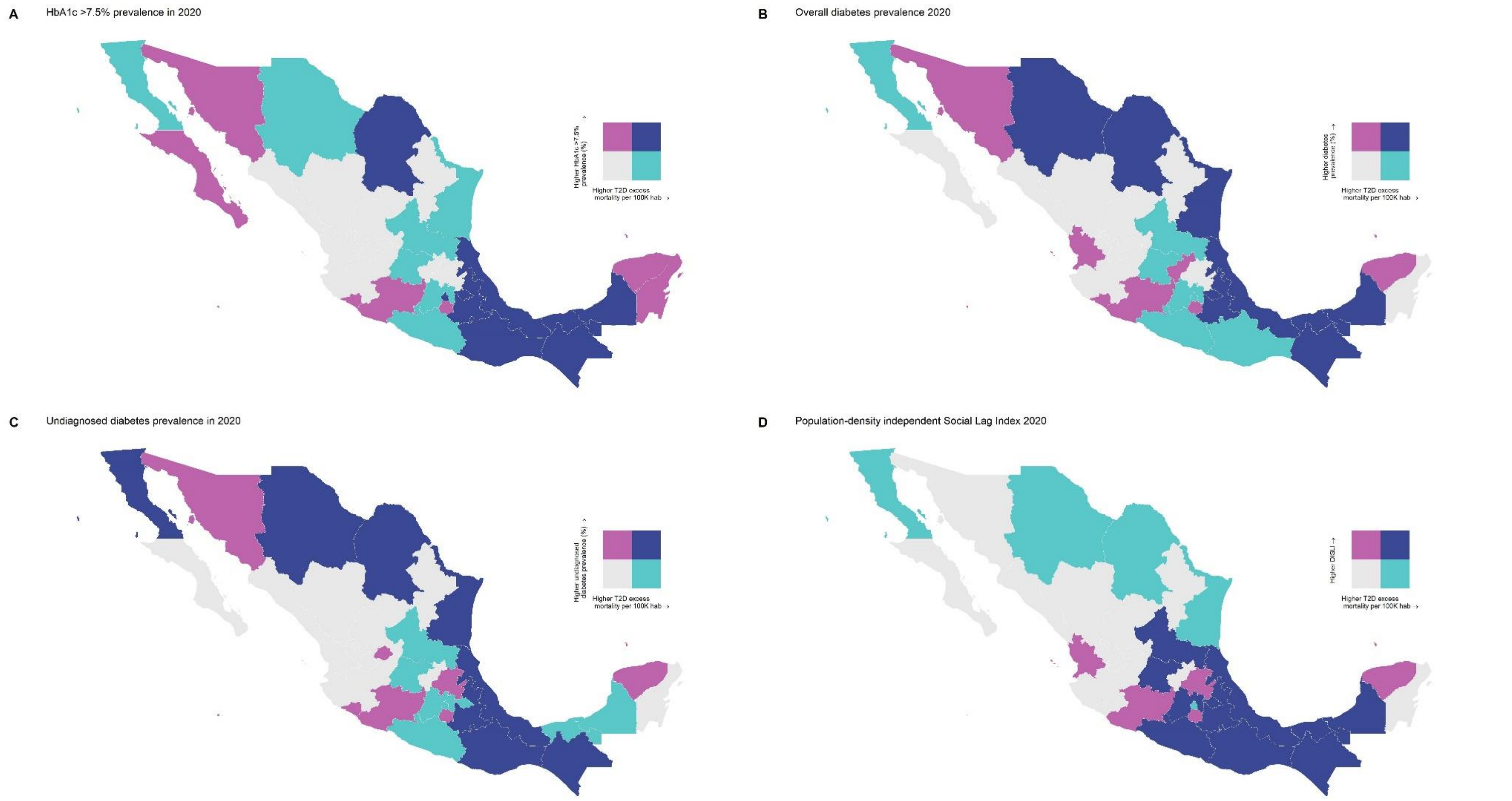

Supplementary Figure 8. Bivariate chloropleth maps showing the geographical distribution of high out-of-hospital to in-hospital diabetes-related mortality in Mexico with epidemiological indicators related to diabetes care including HbA1c >7.5% prevalence (A), overall diabetes prevalence (B), undiagnosed diabetes prevalence (C), and the population-density independent social lag index (DISLI, D). Distribution of all evaluated measures was estimated using the quantile method with the *biscale* R package.

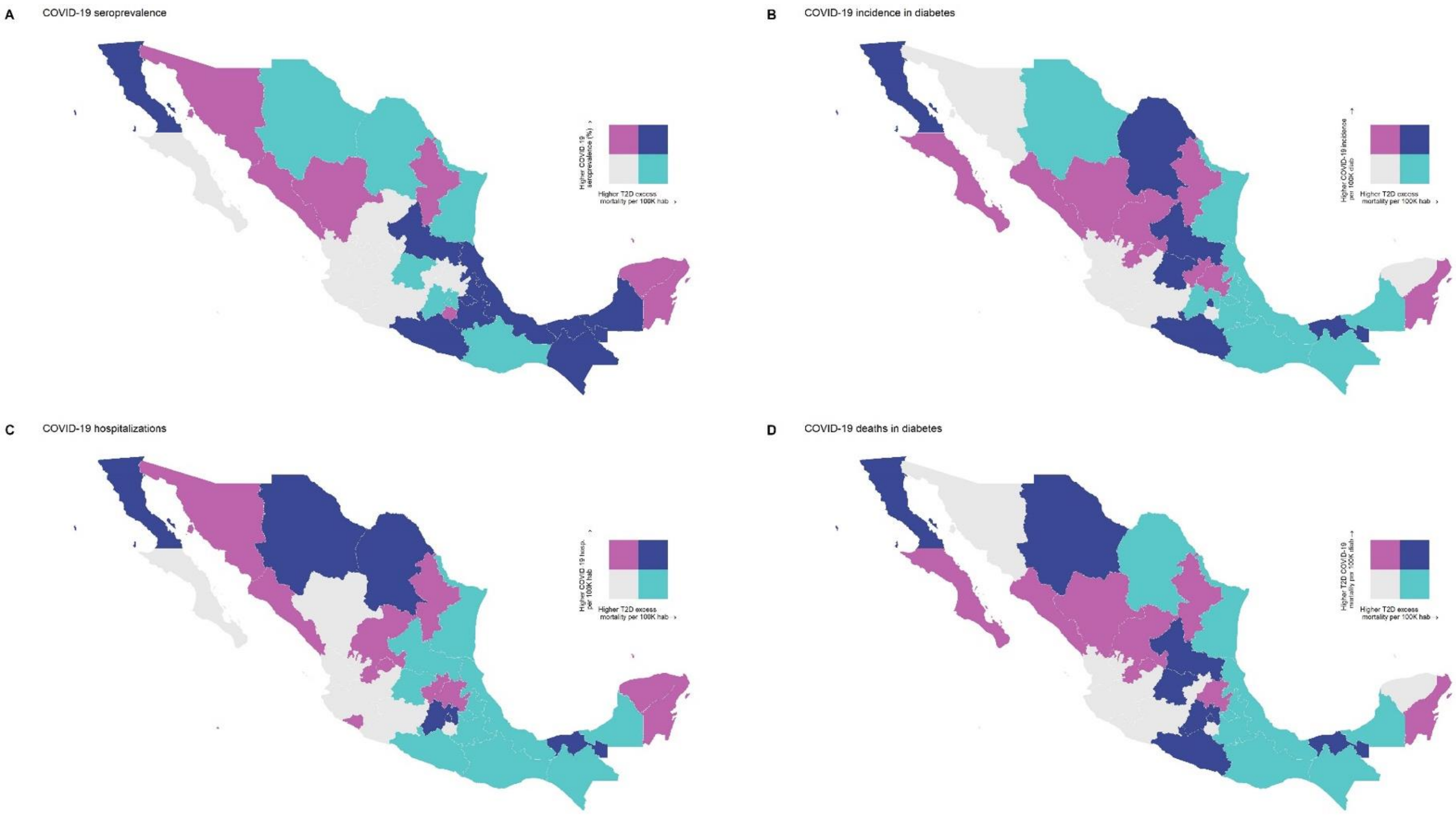

Supplementary Figure 9. Bivariate chloropleth maps showing the geographical distribution of high diabetes-related excess mortality in Mexico with epidemiological indicators related to COVID-19 including COVID-19 seroprevalence (A), COVID-19 incidence in diabetes (B), COVID-19 hospitalizations (C), and COVID-19 deaths in diabetes (D). Distribution of all evaluated measures was estimated using the quantile method with the *biscale* R package.

SUPPLEMENTARY TABLES

Supplementary Table 1. Age-adjusted excess mortality rate per 100,000 habitants in nine Mexican regions during 2020, along with prevalence of diabetes, undiagnosed diabetes, HbA1c ≥7.5% and COVID-19 seroprevalence as estimated using ENSANUT COVID 2020.

Region	Diabetes mortality 2017-2019	Diabetes mortality 2020	Age-adjusted excess mort. rate	Diabetes prev. (%)	Undiagnosed Diabetes (%)	HbA1c ≥7.5% (%)	COVID-19 seroprev. (%)
Mexico City	118.05	178.79	60.74	14.53	2.94	7.15	18.40
Center	137.39	192.68	55.29	22.19	9.56	13.45	23.85
Center-North	122.90	161.14	38.24	12.33	2.50	3.81	17.93
Mexico State	143.74	236.60	92.86	13.24	2.58	6.59	21.99
Frontier	105.59	145.67	40.08	17.05	4.89	6.82	19.73
South pacific	140.49	198.69	58.20	15.91	4.84	7.27	22.76
Center Pacific	117.49	144.78	27.29	14.76	4.48	6.27	18.26
Northern Pacific	91.66	121.24	29.58	14.43	4.62	10.40	28.85
Península	135.41	194.42	59.01	16.77	6.96	11.40	39.74

Abbreviations: Prev., prevalence; seroprev., seroprevalence.

Supplementary Table 2. Negative binomial regression models to assess association of epidemiological indicators of age-adjusted diabetes-related excess mortality stratified by diabetes type. **Abbreviations:** DISLI, Density-independent social lag index; IRR, Incidence Rate Ratio; 95%CI, 95% Confidence interval.

Model	Parameter	IRR (95%CI)	p-value
Overall R^2=0.30	Intercept	3.78 (0.66-21.51)	0.13
	DISLI	1.18 (1.01-1.37)	0.03
	COVID-19 hospitalization	1.28 (1.06-1.55)	0.01
	Prevalence of HbA1c ≥7.5%	1.04 (1.01-1.07)	0.03
Type 2 diabetes R^2=0.23	Intercept	2.88 (0.29-29.03)	0.37
	DISLI	1.32 (1.08-1.63)	0.01
	COVID-19 hospitalization	1.32 (1.01-1.71)	0.04
Other diabetes types R^2=0.20	Intercept	4.90 (2.33-10.29)	<0.01
	COVID-19 seroprevalence	1.02 (1.01-1.04)	0.02
	Diabetes prevalence	1.04 (1.01-1.07)	0.03
Type 1 diabetes R^2=0.07	Intercept	1.71 (0.80-3.62)	0.16
	DISLI	1.00 (0.97-1.03)	0.84
	Diabetes prevalence	0.80 (0.57-1.12)	0.20

REFERENCES

1. Índice Rezago Social 2015. Accessed December 14, 2021. https://www.coneval.org.mx/Medicion/IRS/Paginas/Indice_Rezago_Social_2015.aspx

2. Antonio-Villa NE, Fernandez-Chirino L, Pisanty-Alatorre J, et al. Comprehensive evaluation of the impact of sociodemographic inequalities on adverse outcomes and excess mortality during the COVID-19 pandemic in Mexico City. *Clin Infect Dis Off Publ Infect Dis Soc Am*. Published online June 22, 2021:ciab577. doi:10.1093/cid/ciab577

3. Basto-Abreu AC, López-Olmedo N, Rojas-Martínez R, et al. Prevalence of diabetes and glycemic control in Mexico: national results from 2018 and 2020. *Salud Pública México*. 2021;63(6, Nov-Dic):725-733. doi:10.21149/12842

4. Sánchez-Pájaro A, Ferrer CP, Basto-Abreu A, et al. Seroprevalencia de SARS-CoV-2 en adultos y adultos mayores en México y su asociación con enfermedades crónicas. Ensanut 2020 Covid-19. *Salud Pública México*. 2021;63(6, Nov-Dic):705-712. doi:10.21149/13163

5. Bello-Chavolla OY, Bahena-López JP, Antonio-Villa NE, et al. Predicting Mortality Due to SARS-CoV-2: A Mechanistic Score Relating Obesity and Diabetes to COVID-19 Outcomes in Mexico. *J Clin Endocrinol Metab*. 2020;105(8):2752-2761. doi:10.1210/clinem/dgaa346

6. Bello-Chavolla OY, González-Díaz A, Antonio-Villa NE, et al. Unequal Impact of Structural Health Determinants and Comorbidity on COVID-19 Severity and Lethality in Older Mexican Adults: Considerations Beyond Chronological Aging. *J Gerontol Ser A*. 2021;76(3):e52-e59. doi:10.1093/gerona/glaa163

7. Bello-Chavolla OY, Antonio-Villa NE, Fernández-Chirino L, et al. Diagnostic performance and clinical implications of rapid SARS-CoV-2 antigen testing in Mexico using real-world nationwide COVID-19 registry data. *PLOS ONE*. 2021;16(8):e0256447. doi:10.1371/journal.pone.0256447
